# Patients satisfaction with healthcare delivery in Ghana

**DOI:** 10.1186/s12913-021-06717-5

**Published:** 2021-07-22

**Authors:** Daniel Adjei Amporfro, Michael Boah, Shao Yingqi, Therese Martin Cheteu Wabo, Miaomiao Zhao, Victorine Raissa Ngo Nkondjock, Qunhong Wu

**Affiliations:** 1 Department of Social Medicine, School of Health Management, Harbin Medical University, Harbin, Heilongjiang China; 2 Department of Epidemiology, Biostatistics and Disease Control, School of Public Health, University for Development Studies, Tamale, Ghana; 3 Department of Nutrition and Food Hygiene, Harbin Medical University, Harbin, Heilongjiang China; 4 Department of Health Management, School of Public Health, Nantong University, Nantong, Jiangsu China

**Keywords:** Patient satisfaction, Service quality, Health service delivery, Ghana, Service dimensions, Total satisfaction

## Abstract

**Background:**

The service industry has been an evolving sector and a great concern to providers ensuring continuously that clients’ satisfaction is met. Hence, the importance of patient satisfaction in the healthcare sector. This study focused on the satisfaction of women with the delivery of health services in Ghana and aims to be different from other studies which has focused on patient satisfaction with urban and rural health services, regional health services and health insurance. Our study examines the percentages of satisfaction with the multiple outcomes defined and identifies the key health system and demographic related factors associated with women satisfaction.

**Methods:**

This study used data from the 2014 Ghana Demographic and Health Survey and a total of 12,831 households were systematically selected with reproductive women aged 15–49 years eligible for interview. Data for this study was analysed quantitatively using descriptive statistics, chi square and regression analysis. A total of 3648 women were included in this study and the final analysis thus involved a weighted sample of 3507 women. Satisfaction indicators were put together into SERVQUAL dimensions in the study and reliability test run using Cronbach Alpha (α). All data analyses were carried out in STATA 13.0. The adjusted odds ratios (AOR) with their corresponding 95% confidence intervals (CI) were calculated.

**Results:**

Analysis showed that independently, education and religion were significantly associated with service reliability, overall satisfaction and responsiveness. Payment option was also associated with responsiveness and tangibility dimensions. Furthermore, place of residence was independently associated with responsiveness, tangibility and overall satisfaction. Finally, maternal age, region, provider friendly, ease of getting care and opening hours were all independently associated with reliability, responsiveness, tangibility and overall service satisfaction at the multivariable level.

**Conclusions:**

Dimensions of service quality which focus on patient-centered atmosphere and efficient service delivery system should be integrated and strengthened by hospital management in order to increase patient satisfaction. Key maternal characteristics and health system related factors were revealed to have positive association with patient satisfaction with health services delivery and this cannot be ignored by health care managers in ensuring that systems are improved for better health care.

## Background

Patient satisfaction occurs when healthcare providers exceed their expectation in the delivery of quality health services [1]. This is a key determinant of quality in healthcare delivery and as an indicator which is accepted worldwide, it must continuously be part of standards for institutions and training [2]. Although Gronroos mentioned in their studies that a lot of research has been undertaken in the health sector and the key underlying definition of what health is about was not stated [3, 4], the concept of health as well-being was introduced in the World Health Organization’s (WHO, 1948) definition of health as “a state of complete physical, mental, and social well-being and not merely the absence of disease or infirmity [5].” This definition incorporated multiple dimensions of health, including physical (structure and function), mental (emotional and intellectual), social, role, and general views of health status, paving the way for a paradigm shift from a narrow concentration on physical components of health [6]. Different definitions and types of framework used to measure the quality of care were developed and this has been measured from different perspectives like the population, individual, structure, process and outcome of care. For example, The Institute of Medicine (USA) defines healthcare quality mainly in technical terms as the extent to which health services delivered for the population and especially patients increases the health outcomes consistent with modern knowledge [7]. Ovretveit and Donabedian, and frameworks from other authors have been used to support and to improve the quality of healthcare delivery [8–10]. The quality of healthcare and for that matter healthy population can be achieved if the healthcare provided is of highest standard, universal, affordable and to a larger population of the community [11]. Perceived service quality as explained by Zeithaml is based on the decisions formed by customers of the expected services of the healthcare provider [12]. The actual service delivered by providers confirms the perception of its patients and this could be positive confirmation or disconfirmation. Different elements leading to patient expectation includes adequate service, desired service, tolerance and predicted service that falls between expected service and adequate service levels [13]. Service dimensions as propounded by Parasuraman et al. [14, 15] details the areas of service satisfaction dimensions and have also been cited by other authors under characteristics of service marketing [16] ensuring service providers deliver to the best standards available. Different authors over the past years have identified different service quality dimensions [17] which are adapted for its use depending on the environment and the purpose for which it is being used: as Servqual model cannot be a straight jacket to fit all purpose.

In some of the developed world, being patient-focused is imperative as patient satisfaction plays crucial role in healthcare delivery and the quality of care reforms implemented. Several studies mentioned that satisfied patients are likely to be loyal to their providers and build good relationship with the health system which eventually leads to improved services, loyalty and high satisfaction rate [8, 12, 18–20]. One of the importance of satisfaction is paying attention to feedback from patients who visit various healthcare facilities as it leads to improvement on the care delivered, effectiveness and efficiency of the provider and overall patient satisfaction [19]. It is also suggested that in the context of healthcare, providers must be concerned and ensure eliminating dissatisfaction drivers and focusing on satisfaction [21].

In most developing countries, patients’ satisfaction has been remarkably low although there are several published reports of how quality care in these countries can be delivered. Healthcare facilities in Africa have to be patient-centred and focus on service marketing to ensure sustainability of their services. Maintaining high standards and improving service quality in healthcare in Africa is central to patient satisfaction [22]. Whilst a lot of studies in Africa have implemented customer satisfaction surveys, the factors that precisely satisfy patients are mostly unidentified [23] and therefore organisations are called upon by key stakeholders to pay critical attention to patients feedback [19].

Until recently, there has been a rapid growth of the economy of Ghana and an increase in spending on healthcare [24] as healthcare in Ghana is mainly financed by the government, development partners and households [25]. The delivery of quality care in Ghana has brought key competition between the private and public health institutions by placing service quality first in health policy. The health sector in Ghana must focus on service quality and patient satisfaction because previous studies from government health facilities over a period revealed that healthcare quality in Ghana is not good enough and up to standard and these are opinions of patients and healthcare providers [26, 27]. Based on reports (GHS), healthcare providers are not satisfactorily following the quality care guide agreed on by themselves and are perceived as unresponsive by patients [28]. To make healthcare accessible to the populace and to improve on the quality of care, a strategic plan on quality was put together to help guide health providers highlighting on health status and health services in Ghana and the status of the quality of health. Some of the key objectives in the plan were to (1) improve on client focused services due to bad client relations in public health institutions, (2) improve on patient safety as this is a key part of quality care and intended to prevent medical errors, hence ensuring the safety of healthcare (3) improve clinical practise through activities that result in a patient’s ailment being correctly diagnosed and successfully treated and finally (4) improve management systems through managerial processes used to improve the quality of clinical care in Ghana [28]. Poor quality healthcare can result in loss of patient lives, revenue, time and resources, trust and respect, community apathy and negative hospital reputation [29].

Yaya et al. and Ama Pokuaa F. researched into patient satisfaction in Ghana examining the urban and rural difference in patients satisfaction and patient satisfaction with health insurance (Insured and Non-insured) respectively [8, 30]. Atinga R. researched into patient satisfaction but in the two northern regions of Ghana and there was also an empirical study to assess perceived service quality in hospitals using the service quality model (SERVQUAL) as this can be directly linked to patient satisfaction [26, 31]. Furthermore, Dzomeku, M. did a study on maternal satisfaction with care during labour in a district hospital in Ghana [32]. Our study was conducted with the focus on satisfaction of women (patients) with the delivery of health services in facilities in Ghana. The study focused on women because most prior global studies found out that women rely on medical services more than men and also have a different anatomical function from men [33, 34]. Secondly, health utilisation of men in this study was very low and in comparing the sample size of both genders, it was therefore not appropriate to combine with women. Different studies from Ghana have focused on patient satisfaction as a general outcome [35–37] but our study focused on multiple outcomes and total service satisfaction. Our main objectives, therefore of the study were 1. To explore the association of key independent factors with the multiple outcomes of the study and 2. Examine satisfaction level with the three-dimensional areas of service delivery namely responsiveness, reliability and tangibility and overall satisfaction.

## Methodology

This study used data from the 2014 Ghana Demographic and Health Survey. The Ghana Statistical Service (GSS), Ghana Health Service (GHS) and the GHS National Public Health Reference Laboratory (NPHRL) implemented the 2014 GDHS survey. The survey is nationally representative and provides data on key health indicators including fertility, reproductive health, child health and nutrition. A multi-stage sampling method was used to select clusters and households for the survey. A total of 12,831 households were systematically selected using a sampling frame that was updated from the 2010 population and housing census (PHC) in Ghana. All reproductive women aged 15–49 years who were permanent residents or visitors who passed the night preceding the survey in a selected household were eligible for interview. Household demographic data and information on fertility, morbidity, child health and mortality were gathered using three questionnaires. The response rate was 97% for the women’s questionnaire. Detailed information on the 2014 GDHS survey design and data collection process as well as the questionnaire used can be found in the final report (Ghana Statistical Service, Ghana Health Service, & ICF International, 2015).

Due to the focus of the present study, the analysis was restricted to only women who used health care services in the past 6 months preceding the GDHS survey. A total of 3648 women were included in this study. However, due to the complexity of the design used by the Demographic and Health Survey (DHS), weights had to be applied to adjust for the disproportionate sampling (Demographic and Health Survey, 2006). The final analysis thus involved a weighted sample of 3507 women.

### Variables

#### Dependent variables

Four dependent variables, including service reliability, service responsiveness, service tangibles, and total satisfaction were selected for the present study. Service reliability was defined in the present study as the consistency of service performance and trustworthiness in delivering health services right the first time. It also refers to the fulfilment of the promises of care by the provider to the patient/public by a reasonable and definite time avoiding or reducing errors to the barest minimum. Tangibles in this study refers to the physical appearance of the health facility, the atmosphere in which care is delivered and cleanliness of the facility. It also refers to infrastructure, equipments, seats and facility signage. Responsiveness refers to the readiness of staff to provide service to patients within the required timelines by responding to patients and delivering care and lastly, total satisfaction is the combination of the three service dimensions above to make a fourth dependent variable. The interaction, diagnosis and treatment given defines the moment of truth between patient and service provider. Service reliability was derived from responses, which measured patients’ satisfaction with the following: waiting time to wait for turn, time spent in examination/consulting room, time spent for tests to be performed, time to wait for test results, and waiting time at pharmacy/dispensary. Service responsiveness was derived from responses on patients’ satisfaction with privacy during examination, staff listened to patient, staff explained to patient what she wanted, staff advised patient on treatment, provider spent enough time with patient, provider sought consent before treatment, and confidentiality and protection of personal information. Tangibility was measured using responses on patients’ satisfaction with the cleanliness of the health facility, safety and comfort while waiting, and ease of finding where to go. In the GDHS dataset, responses on client satisfaction were either in binary form (i.e. yes or no) or five ranked responses (i.e. very satisfied, satisfied, fairly satisfied, not satisfied, and very dissatisfied). Binary responses were generated from the five-ranked scale responses by combining very satisfied, satisfied, and fairly satisfied into one outcome, “satisfied” and not satisfied and very dissatisfied into one outcome, “not satisfied”.

In order to measure total satisfaction with service delivery, a dummy variable was created. A score of “0” points was assigned to a “No” response and a score of “1” point was assigned to a “Yes” response. The scores of the individual variables under service reliability, responsiveness, and tangibles were summed and the mean score calculated. A total score below the mean score was classified as not satisfied while a score equal or more than the mean score was classified as satisfied.

#### Independent variables

The independent variables included in the study included both demographic and health system related factors. These factors were selected based on their known association with the dependent variable [38]. They included maternal age, education, religion, region of residence, place of residence (urban or rural), employment status, household wealth index, payment option, service type, facility type, ease of getting care, provider friendliness, and opening hours of facility. The details can be found in Table 1.

**Table 1:** Background characteristics of the women included in the present study

Variable	Frequency	Percentage
**Age**		
15–25	1103	31.5
26–35	1314	37.5
36–49	1090	31.0
**Highest level of education**		
No formal education	688	19.6
Basic	554	15.8
Secondary	1959	55.9
Tertiary	306	8.7
**Religion**		
Traditional	140	4.0
Islam	597	17.0
Christian	2770	79.0
**Region**		
Western	324	9.2
Central	285	8.1
Greater	682	19.4
Volta	295	8.4
Eastern	330	9.4
Ashanti	703	20.0
Brong Ahafo	335	9.6
Northern	279	8.0
Upper East	169	4.8
Upper West	105	3.0
**Place of residence**		
Urban	1958	55.8
Rural	1549	44.2
**Employment**		
Unemployed	674	19.2
Employed	2833	80.8
**Wealth index**		
Poorest	543	15.5
Poorer	560	16.0
Middle	725	20.7
Richer	790	22.5
Richest	889	25.3
**Payment option**		
Cash	1004	28.6
Insurance	2240	63.9
Cash & Insurance	263	7.5

### Data analysis

All data analyses were carried out in STATA 13.0 for Windows (StataCorp LP, College Station, Texas USA). The “svy” command prefix was used in the analysis to account for the study design used by the DHS program. Pearson design-based Chi-square test was used to explore the association between each independent variable and each of the dependent variables. All the independent variables were simultaneously fitted in multivariable logistic regression model. The adjusted odds ratios (AOR) with their corresponding 95% confidence intervals (CI) were calculated. A *p-*value less than 0.05 was considered statistically significant in the final model.

## Results

### Reliability statistics

The reliability of the variables measuring the dependent variables was assessed using Cronbach’s alpha (See Table 2).

**Table 2:** The reliability statistics calculated for the service dimensions

SERVQUAL Dimensions	Number Of Items	Cronbach’s Alpha Value
Reliability	5	0.828
Responsiveness	7	0.696
Tangibles	3	0.505
Total Satisfaction	15	0.801

This indicates that the Cronbach’s alpha values for the measurement sets used in the study for service reliability and total satisfaction are above the recommended value of 0.70, [39]. Although the values for service responsiveness and service tangibility did not meet the recommended value, it can therefore be concluded that two of the dimensions were in fact reliable and two were not.

### Chi - square

Pearson design-based Chi-square test was used to examine the association between key explanatory variables with the dimensions of service satisfaction. The results have been presented in Table 3.

**Table 3:** Univariate analysis of factors associated with service reliability, responsiveness, tangibles, and overall satisfaction with health services

Variable	Weighted percentage of respondents who reported being satisfied							
	Reliability	***P-value***	Responsiveness	***P-value***	Tangibles	***P-value***	Total satisfaction	***P-value***
**Age**		0.018		0.073		0.004		0.013
15–25	56.4		75.7		94.2		59.4	
26–35	59.2		79.2		95.0		62.2	
36–49	64.2		81.1		97.3		67.1	
**Highest level of education**		0.049		0.214		0.010		0.052
No formal education	64.2		75.9		96.9		67.2	
Basic education	63.4		83.2		97.3		65.6	
Secondary or higher	57.6		78.7		94.7		60.8	
Tertiary	58.0		76.9		93.4		61.5	
**Religion**		0.643		0.002		0.289		0.584
African traditional	61.1		88.5		96.3		68.1	
Islam	61.9		69.9		96.9		62.7	
Christian	59.4		80.1		95.1		62.6	
**Region**		< 0.001		< 0.001		0.001		< 0.001
Greater Accra	58.7		68.3		93.1		61.7	
Western	81.4		83.7		96.0		83.0	
Central	68.7		80.1		95.8		69.8	
Volta	56.3		79.0		94.8		61.7	
Eastern	55.8		85.4		95.2		62.3	
Ashanti	52.4		82.4		97.2		55.0	
Brong Ahafo	56.6		88.6		98.2		59.1	
Northern	54.5		54.8		91.2		57.7	
Upper East	71.0		92.7		97.9		71.8	
Upper West	57.2		90.5		97.9		58.9	
**Place of residence**		0.112		0.001		0.016		0.005
Urban	58.1		74.1		94.6		59.8	
Rural	62.2		84.5		96.6		66.8	
**Employment**		0.573		0.577		0.833		0.886
Unemployed	58.7		77.8		95.3		62.6	
Employed	60.2		78.9		95.5		62.9	
**Wealth index**		0.166		0.025		0.534		0.334
Poorest	60.7		79.3		96.0		64.0	
Poorer	56.7		84.1		96.1		62.0	
Middle	64.1		82.4		94.8		66.0	
Richer	61.7		77.3		96.3		63.8	
Richest	56.4		73.2		94.5		59.3	
**Payment option**		0.133		0.250		< 0.001		0.362
Cash	59.9		79.3		92.8		62.8	
Insurance	60.8		79.2		96.4		63.5	
Cash & Insurance	52.1		71.9		97.3		57.7	
**Service type**		0.967		0.675		0.265		0.660
Outpatient	59.8		79.6		95.7		61.8	
Inpatient	59.9		78.5		94.5		63.1	
**Type of facility (*N*** **= 3507)**		0.185		0.527		0.178		0.386
Public	59.2		78.3		95.2		62.4	
Private	62.7		80.1		96.6		64.7	
**Ease of getting care**		< 0.001		< 0.001		< 0.001		< 0.001
Not easy	20.5		47.4		83.0		27.2	
Easy	62.2		80.5		96.2		64.9	
**Provider friendliness**		< 0.001		< 0.001		< 0.001		< 0.001
Not friendly	33.2		21.0		81.1		26.4	
Friendly	61.4		81.9		96.3		64.9	
**Opening hours**		< 0.001		< 0.001		< 0.001		< 0.001
Poor	8.8		39.1		76.5		17.2	
Fair	46.7		67.3		93.0		53.4	
Good	62.7		80.9		96.3		65.2	

*p-*value < 0.05 was considered statistically significant in the final

### Satisfaction with service reliability

The univariate analysis showed that women’s satisfaction with service reliability differed according certain demographic and health system related factors, including maternal age, education, region, ease of getting care, provider friendliness, and opening hours. From the findings, higher likelihood of reporting satisfaction with service reliability was observed among women in the following categories; age range of 36–49 years, no formal education, western region, easy getting care, provider friendly, and good opening hours of health facility (Table 3).

### Satisfaction with service responsiveness

The results showed variations in reporting satisfaction in service responsiveness with regards to maternal age, religion, region, place of residence, wealth group, ease of getting care, provider friendliness, and opening hours of health facility. Comparatively, women in the age group of 36–49 years (81.1%); African traditional religion (88.5%); from Upper East Region (92.7%); rural women (84.5%); and poorer women (84.1%). It was also noted that women who found it easy getting care (80.5%), the provider friendly (81.9%), and the opening hours good (80.9%) were more likely to report being satisfied with service responsiveness (Table 3).

### Satisfaction with service tangibles

The results showed that demographic factors, including maternal age, education, region, and place of residence were found to be significantly associated with women’s report of satisfaction with service tangibles. Regarding the health system related factors, the following factors, including payment option, ease of getting care, provider friendliness, and opening hours were identified as statistically significant correlates of satisfaction with service tangibles (Table 3).

### Overall service satisfaction

The maternal age, region, place of residence, ease of getting care, provider friendliness, and opening hours were the factors found to be associated with overall service satisfaction in the univariate analysis. The results showed that women in the age group of 36–49 years (67.1%), western region (83.0%), and rural women (66.8%) were significantly more likely than their colleagues to report being satisfied with the overall services they received in their most recent visit to the health facility. Likewise, women who found it easy to get care (64.9%), provider friendly (64.9%), and good opening hours (65.2%) were more likely than their counterparts to report being satisfied with the overall services received (Table 3).

The independent association of the explanatory factors with the three dimensions of service satisfaction as well as overall service satisfaction was evaluated. The results showed that maternal age and region were independent demographic factors associated with satisfaction with service reliability. Regarding the health service-related factors, ease of getting care, provider friendliness, and opening hours were found to be correlated with satisfaction. Comparatively, higher odds of reporting satisfaction with service reliability were noted among women in the age group of 36–49 years (AOR = 1.36; 95% CI: 1.07–1.72) and women in the western region (AOR = 3.14; 95% CI: 1.86–5.30). Similarly, women who reported that it was easy to get care (AOR = 4.20; 95% CI: 2.76–6.40), provider was friendly (AOR = 2.53; 95% CI: 1.68–3.82), and opening hours of the facility were fair (AOR = 7.11; 95% CI: 3.11–16.23) or good (AOR = 12.53; 95% CI: 5.66–27.72) had higher odds of being satisfied with service reliability compared with their counterparts (Table 4).

**Table 4:** Independent demographic and health services-related factors associated with patient satisfaction

Variable	Reliability	***P***-value	Responsiveness	***P***-value	Tangibles	***P***-value	Total satisfaction	***P***-value
**Age**								
15–25	Ref							
26–35	1.09 (0.88–1.36)	0.438	1.43 (1.07–1.91)	0.015	1.03 (0.62–1.70)	0.907	1.10 (0.88–1.38)	0.381
36–49	1.36 (1.07–1.72)	0.011	1.44 (1.04–2.00)	0.028	1.84 (1.01–3.37)	0.046	1.36 (1.08–1.71)	0.008
**Highest level of education**								
No formal education	Ref							
Basic education	0.90 (0.66–1.22)	0.492	1.19 (0.76–1.87)	0.452	1.16 (0.50–2.72)	0.730	0.84 (0.62–1.15)	0.278
Secondary	0.77 (0.59–1.00)	0.051	1.21 (0.82–1.78)	0.342	0.49 (0.22–1.11)	0.086	0.76 (0.58–0.99)	0.044
Tertiary	0.78 (0.51–1.25)	0.320	1.29 (0.71–2.37)	0.404	0.45 (0.14–1.42)	0.175	0.80 (0.52–1.23)	0.304
**Religion**								
African traditional	Ref							
Islam	1.20 (0.73–1.97)	0.474	0.30 (0.14–0.66)	0.003	1.91 (0.38–9.59)	0.430	0.95 (0.56–1.59)	0.831
Christian	096 (0.62–1.48)	0.848	0.40 (0.19–0.83)	0.014	0.90 (0.22–3.68)	0.886	0.82 (0.51–1.32)	0.417
**Region**								
Greater Accra	Ref							
Western	3.14 (1.86–5.30)	< 0.001	2.25 (1.22–4.14)	0.009	1.60 (0.71–3.62)	0.260	2.96 (1.81–4.86)	< 0.001
Central	1.41 (0.86–2.31)	0.168	1.41 (0.76–2.62)	0.268	2.23 (0.90–5.49)	0.081	1.23 (0.77–1.98)	0.388
Volta	1.10 (0.67–1.80)	0.700	2.19 (1.20–4.00)	0.011	1.28 (0.51–3.22)	0.605	1.14 (0.69–1.88)	0.600
Eastern	0.88 (0.56–1.39)	0.581	2.46 (1.37–4.40)	0.003	1.19 (0.50–2.81)	0.696	0.94 (0.58–1.51)	0.791
Ashanti	0.78 (0.51–1.18)	0.237	2.64 (1.48–4.70)	0.001	2.56 (1.04–6.31)	0.041	0.72 (0.48–1.08)	0.114
Brong Ahafo	0.83 (0.53–1.30)	0.422	3.29 (1.69–6.38)	< 0.001	3.12 (1.02–9.52)	0.045	0.78 (0.51–1.21)	0.266
Northern	0.76 (0.45–1.29)	0.317	0.56 (0.29–1.08)	0.082	0.26 (0.09–0.79)	0.017	0.74 (0.44–1.23)	0.246
Upper East	1.44 (0.86–2.41)	0.163	4.96 (2.45–10.05)	< 0.001	1.29 (0.32–5.25)	0.720	1.24 (0.76–2.00)	0.392
Upper West	0.79 (0.36–1.75)	0.559	4.99 (2.19–11.33)	< 0.001	1.03 (0.28–3.81)	0.965	0.69 (0.35–1.35)	0.277
**Place of residence**								
Urban	Ref		Ref					
Rural	1.15 (0.86–1.54)	0.341	1.88 (1.30–2.72)	0.001	1.30 (0.77–2.19)	0.322	1.38 (1.05–1.81)	0.020
**Employment**								
Unemployed	Ref							
Employed	0.96 (0.77–1.21)	0.730	0.89 (0.67–1.18)	0.424	0.76 (0.41–1.40)	0.372	0.91 (0.71–1.15)	0.414
**Wealth index**								
Poorest	Ref							
Poorer	0.83 (0.58–1.20)	0.325	1.12 (0.78–1.60)	0.552	0.56 (0.21–1.45)	0.231	0.89 (0.61–1.30)	0.547
Middle	1.18 (0.83–1.70)	0.355	1.10 (0.69–1.76)	0.696	0.62 (0.24–1.61)	0.321	1.15 (0.81–1.64)	0.429
Richer	1.17 (0.74–1.82)	0.502	0.96 (0.58–1.59)	0.874	0.72 (0.23–2.22)	0.566	1.22 (0.79–1.88)	0.375
Richest	0.96 (0.57–1.63)	0.892	0.87 (0.50–1.53)	0.633	0.70 (0.19–2.60)	0.595	1.07 (0.65–1.75)	0.790
**Payment option used**								
Cash	Ref							
Insurance	0.99 (0.79–1.26)	0.957	0.71 (0.51–0.99)	0.049	2.00 (1.25–3.18)	0.004	0.98 (0.77–1.23)	0.838
Cash & Insurance	0.95 (0.65–1.38)	0.768	0.55 (0.33–0.93)	0.026	2.78 (1.07–7.25)	0.036	1.06 (0.73–1.53)	0.761
**Service type**								
Outpatient	Ref							
Inpatient	1.04 (0.81–1.34)	0.743	1.06 (0.75–1.49)	0.741	0.75 (0.41–1.37)	0.347	1.00 (0.79–1.27)	0.993
**Type of facility**								
Public	Ref							
Private	1.08 (0.83–1.39)	0.567	1.08 (0.79–1.48)	0.617	2.02 (1.13–3.63)	0.019	1.04 (0.80–1.33)	0.785
**Ease of getting care**								
Not easy	Ref							
Easy	4.20 (2.76–6.40)	< 0.001	2.50 (1.68–3.72)	< 0.001	2.64 (1.38–5.03)	0.003	3.25 (2.22–4.75)	< 0.001
**Provider friendliness**								
Not friendly	Ref							
Friendly	2.53 (1.68–3.82)	< 0.001	15.45 (9.72–24.56)	< 0.001	3.76 (2.10–6.72)	< 0.001	4.48 (2.95–6.81)	< 0.001
**Opening hours**								
Poor	Ref							
Fair	7.11 (3.11–16.23)	< 0.001	2.61 (1.40–4.88)	0.003	3.20 (1.27–8.03)	0.013	4.15 (2.078–8.31)	< 0.001
Good	12.53 (5.66–27.72)	< 0.001	5.78 (3.35–9.97)	< 0.001	5.38 (2.67–10.84)	< 0.001	6.22 (3.27–11.82)	< 0.001

*p-*value < 0.05 was considered statistically significant in the final

The analysis also found important correlates of service responsiveness. The results showed that among the demographic factors, women aged 36–49 years and women from rural settings had increased odds of reporting being satisfied with service responsiveness (AOR = 1.44; 95% CI:1.04–2.00 and AOR = 1.88; 95% CI:1.30–2.70, respectively). Furthermore, higher odds were reported among women from Western (AOR = 2.25; 95% CI: 1.22–4.14), Volta (AOR = 2.19; 95% CI: 1.20–4.00), Eastern (AOR = 2.46; 95% CI:1.37–4.40), Ashanti (AOR = 2.64; 95% CI:1.48–4.70), Brong Ahafo (AOR = 3.29; 95% CI:1.69–6.38), Upper east (AOR = 4.96; 95% CI:2.45–10.05), and Upper west (AOR = 4.99; 95% CI:2.19–11.33) regions compared with women from Greater Accra region. However, lower odds of reporting satisfaction with service responsiveness were recorded among women of the Islam and Christian religious groups. Regarding the health service related factors, it was found that women who paid for services using insurance and both cash and insurance were less likely to be satisfied with service responsiveness relative to women who paid on cash for services (AOR = 0.71; 95% CI: 0.51–0.99 and AOR = 0.55; 95% CI: 0.33–0.93, respectively). However, women who reported that it was easy to get care, provider was friendly, and the opening hours of the health facility were fair and good, were more likely than their colleagues to be satisfied with service responsiveness (Table 4).

The results showed that maternal age and region were significantly associated with reporting satisfaction with tangibles. In addition, we noted the payment option used, the type of facility visited, the ease of getting care, provider friendliness, and facility’s opening hours, were correlated with reporting satisfaction with service tangibles. For instance, the results showed that women who attended private health facilities were two times more likely than women who attended public health facilities to report being satisfied with service tangibles (AOR = 2.02; 95% CI:1.13–3.63).

Finally, the independent correlates of overall service satisfaction were examined. The findings showed that age, maternal education, region, and place of residence were important demographic factors associated with service satisfaction. The data showed that comparatively, older women (36–49 years), women from western region, and rural women had higher odds of reporting overall satisfaction with services (AOR = 1.36; 95% CI: 1.08–1.71, AOR = 2.96; 95% CI: 1.81–4.86, and AOR = 1.38; 95% CI: 1.05–1.81, respectively). Regarding the health system-related factors, we observed that ease of getting care, provider friendliness, and opening hours were significant factors of overall satisfaction with health services. Women who found it easy to get care were more likely to report being satisfied with services relative to women who did not (AOR = 3.25; 95% CI:2.22–4.75). Similarly, women who found the service provider friendly were four times more likely than women who did not find the provider friendly to report being satisfied with services (AOR = 4.48; 95% CI:2.95–6.81) (Table 4).

## Discussion

**Fig. 1:**
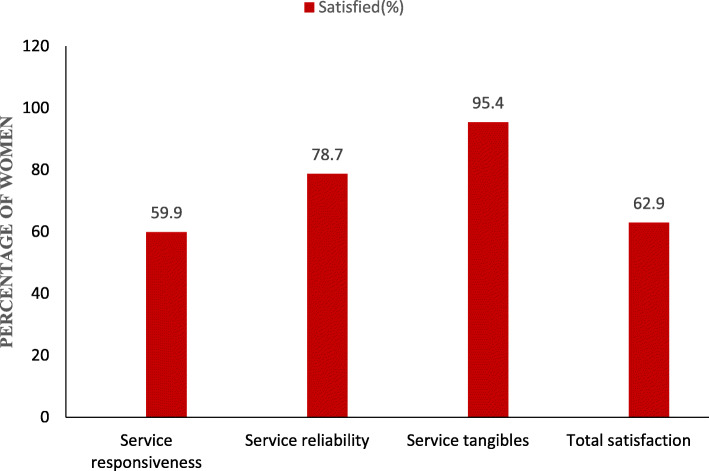
Satisfaction level with service reliability, responsiveness, tangibles, and overall satisfaction with health services. Legend: The results showed that of the total women included in the present study, 59.9% (95% CI: 57.3–62.4), 78.7% (95% CI: 75.8–81.4), 95.4% (95% CI: 94.6–96.2) reported being satisfied with service responsiveness, service reliability, and service tangibles respectively. In general, 62.9% (95% CI: 60.3–65.3) of the total sample was satisfied with the overall services they received during their most recent visit to the health facility. The percentage of satisfaction according the individual areas used to measure the service dimensions is shown in the bar graph

The service quality dimensions and patient satisfaction discussed in this paper opens up ideas for researchers and practitioners that could lead to improvements in service quality and patient satisfaction in hospitals in Ghana. Figure 1 shows the percentages of satisfied patients (women) with the three dimensions of health service delivery and total satisfaction. All of these service dimensions are strictly independent of each other and which explains that irrespective of age, background, status or relationship with a patient, the dimensions must be an important indicator in service measurements and patients must receive prompt services from their providers [40]. To a large extent, it is imperative that providers focus on being responsive with their service delivery as services are delivered by providers whilst consumed by patients. Providing excellent healthcare services to patients is equally important to retain these patients [17]. Satisfaction with service reliability is high which could be attributed to providers paying attention to these areas. The variables comprising reliability indicate and support the reason for high satisfaction rate by patients. This means the patients were attended to by their respective providers for their sickness and received the service they perceived to receive at the facility [40]. Service tangibles had a higher satisfaction rate above 90%. This further illustrates the facility, access areas, standard procedures in place, aesthetics and the atmosphere in which the service is delivered which reinforces patients’ safety and comfortability at the facility [40].

Considering the characteristics of services marketing for further illustration, inseparability in health service delivery means patients come for consumption of services whilst the provider delivers it. There is a perceived and expected service to be received by the patient [16] and anything that falls short leads to dissatisfaction which is reflected in the variance of percentages of satisfaction. Service heterogeneity in health facilities across the country or with same facilities makes it difficult most at times for patients to have same or similar service on a second visit [40] and this results in variation in service satisfaction level by patients. This is because a patient may find him or herself going through different prescribers or departments in the facility [40, 41]; for example the emergency unit, laboratory staff and dispensary and may not have consistent services throughout the visit. The intangibility nature of service delivery makes it important especially access, delivery and outcome of the care and therefore the responsibility lies with the providers to deliver services that exceeds patients expectation and to lower or clear perceived risk [42]. The physical confirmation of tangibility that patients receive could be patient folders, hospital cards, test results, receipts and drugs as a proof of services they have received. It is necessary that the tangibles are in place and appealing and for providers to be responsive and reliable to patients when they report at the facility since care is essential and paid for when it is delivered [43].

Using logistic regression, our results revealed that health system related factors and some of the maternal demographics in the study were positively associated with one or all of the multiple outcomes of the study. Although, some variables were positively associated with waiting time indicators in our study, other studies show that there are always high levels of dissatisfaction with waiting times irrespective of hospitals or countries [44]. Ease of getting care was positively associated with the reliability outcome consisting of indicators like waiting time to be called, time spent to do test and collection of results, time spent for consultation and other indicators. This is in congruent with a study by Panchapakesan et al. who grouped waiting time indicators as administrative procedure dimension and mentioned that public hospitals in India are known to offer word class treatment and have easier procedures for admission [45]. Age was positively associated with the reliability outcome. However, in the study of Westaway et al., age or any other demographics of patients were found not to be related to any of the dimension they discussed. Friendly attitude of personnel at health facilities improves retention rates of patients and also patients trust employees with excellent clinical encounters and manages their private and confidential information well. Provider friendliness was found to influence patient satisfaction and positively associated with the four outcomes of the study [46, 47].

Religion (both Islam and Christian) in our study had a positive association with service satisfaction (responsiveness). The findings on the positive association between the Islamic religion and service satisfaction is in line with another study from Nigeria which could, however not be attributed to any specific reasons. However, it is a known fact that Islamic women will want to be taken care of by women prescribers and other women staff at the facility. Respecting these guidelines can help Muslim patients communicate more honestly with their providers and receive the treatment they need. The immediate services they receive through interactions, diagnosis and treatment and the responsive care given at the facility is likely to influence their satisfaction [48, 49]. A further study is needed to understand the underlying motives or perceived reasons on how their religion influences their satisfaction.

Patients’ age was found to have a positive association with the tangibility dimension of service and not total satisfaction. This in combination with education reveals patients’ maturity and assessment of healthcare delivery [50]. The older the patients, the more likely they are to have substantial experience in dealing with health facilities and this influences their choice of facility and measurements of services delivered. A physical representation of the service patients receive is through the tangible items like insurance card, drugs and the patients’ folder. A very well laid out facility, clean equipment, and availability of tangibles help to significantly improve patients’ perception about quality and their overall satisfaction with provided services [51].

With the overall satisfaction, mothers who are educated are less satisfied with health care services. This is because they are more critical of health services offered to them in general and are more knowledgeable about social health issues [52]. Our study shows positive association of education to overall service satisfaction. This is in congruence with a study from Ghana that explained that being literate is found to influence the assessment of health service delivery as educated patients based their decision on information, processes, experience and procedures [32]. This was also corroborated by a study from Das et al. who mentioned that patients who are uneducated assessed the services to be good as compared to the educated patients who considered the services as poor [53]. Provider friendliness and professional service offered by health facilities lead to higher satisfaction. These factors are modifiable, yet essential determinants of care quality that healthcare managers should consider. A service delivery point’s good reputation often attracts clients who return, which promotes access and utilization [54]. Client satisfaction will be enhanced if they can get the opportunity to have a full interaction on their condition and the required treatment. Other authors state that friendly attitude of providers and personnel at the health facilities improves retention rate of patients, employees trust and with excellent clinical encounters and providers who manages their private and confidential information well [46, 47, 55]. Ease of getting care was positively associated with overall satisfaction of the services. This is consistent with the study of Srivastava et. el where ease of getting care was a combination of access to healthcare and location of facility. Getting immediate access to a provider and when the location of the facility is appropriate to the patient highly influences satisfaction [38]. This can be further explained that patients who live closer to health facilities are able to get faster due to the proximity of the facilities even if their condition is not very good.

The implications of this study are that, it will help policy makers and heads of health institutions to focus on key areas of service delivery and to improve on services to the satisfaction of clients. Although there have been a lot and different studies on service satisfaction and the dimensions of service quality, our study focuses on the satisfaction of reproductive mothers and health services received within a particular period. This will offer knowledge to individuals and institutions to help improve their services. The total satisfaction rate of 62.9% as indicated in the results is considered not very good and that areas of and comments on dissatisfaction will aid management considerably to improve on health services. The dimensions of service quality, which focus on patient-centered environment and efficient service delivery system should be incorporated and strengthened by hospital management in order to increase patient satisfaction. By gaining a greater understanding and evaluation of patient satisfaction, health care providers and managers can provide for a more effective and efficient health care system.

This study revealed key health system related and demographic factors associated with service reliability, responsiveness, tangibility and overall service satisfaction. However, to have a stronger justification for the satisfaction of patients for policy making and/or reforms in the health sector, future surveys must have broader criteria and satisfaction indicators to include more men. Furthermore, private facilities must be separated from public facilities as their nature, size and control influences satisfaction and to have a reason for patients’ choice of visiting both facilities. Qualitative studies will further explore the reasons why patients were satisfied or dissatisfied with healthcare delivery at facilities visited. A further study with health providers about patients’ satisfaction and dissatisfaction is needed to help make new policies and reforms to bridge the gap of perception and the service actually delivered.

This current study has its strengths and limitations. One strength of this study is that it used a nationally representative sample and the analysis adjusted for the disproportional method of sampling used by the DHS in its surveys, thereby ensuring that the estimates are reliable and the findings can be generalized to women in the reproductive age group. Also, the GDHS survey involved women recalling events in a five-year period. This predisposes the study to recall bias. However, this analysis was restricted to only women who utilized health services in the past 6 months preceding the GDHS survey. This we believe would minimize recall bias. Furthermore, the cross-sectional design used for this study makes it difficult to make causal inferences, but only that a number of factors are associated with the outcome. Some determinants, such as availability/adequacy of human resources, medicine, supplies and emotional support are known to be associated with satisfaction but are not explored in this study because information was not available in the dataset. Lastly, we collapsed a five-level likert scale responses into binary form to achieve our dependent variables. We recognize as a limitation that we may have lost the “granularity” in the process. Furthermore, the authors used Cronbach’s alpha test for reliability of the satisfaction scales, which showed that two of the service dimensional areas, which were included for analysis namely, responsiveness and tangibility did not meet the reliability test. It can therefore be concluded that the two dimensions were in fact not reliable. Nevertheless, the aim of the present study was to explore the factors associated with reporting satisfaction regardless of the level or degree of satisfaction. Therefore, by collapsing the variables, the data still served this purpose. Moreover, the likert scale did not have a “neutral response” group, thus, respondents either reported some level of satisfaction or some level of dissatisfaction.

## Conclusion

In conclusion, the study revealed key maternal characteristics and health system related factors that have positive association with patient satisfaction with health services delivery in Ghana. Fifteen satisfaction indicators in the study were grouped into different service dimensional areas namely service responsiveness, service reliability, service tangibles and all put together as total service satisfaction. This explored the percentage levels of patients satisfied with the multiple outcomes although two of the outcomes were not reliable. Almost all of the health system related factors had positive association with the outcomes of satisfaction and this cannot be ignored by health care managers in ensuring that systems are improved for better health care. The report of this study is very relevant and important in the delivery of maternal healthcare as health institutions focusing on maternal services will have a plethora of knowledge to identify service gaps and to recommend interventions to improve their services. There are other indicators which are also very important in measuring satisfaction in health care delivery which can be included in future studies. There should be a continuous process in monitoring and evaluating health care services and using patient feedback and research information to create new policies and reforms and to improve and deliver excellent health care.

## Data Availability

The dataset supporting the conclusions of this study is publicly available at www.dhsprogram.com/data/available-datasets.cfm.
