# Correction to: Patients satisfaction with healthcare delivery in Ghana

**DOI:** 10.1186/s12913-021-06894-3

**Published:** 2021-08-25

**Authors:** Daniel Adjei Amporfro, Michael Boah, Shao Yingqi, Therese Martin Cheteu Wabo, Miaomiao Zhao, Victorine Raissa Ngo Nkondjock, Qunhong Wu

**Affiliations:** 1 Department of Social Medicine, School of Health Management, Harbin Medical University, Harbin, Heilongjiang China; 2 Department of Epidemiology, Biostatistics and Disease Control, School of Public Health, University for Development Studies, Tamale, Ghana; 3 Department of Nutrition and Food Hygiene, Harbin Medical University, Harbin, Heilongjiang China; 4 Department of Health Management, School of Public Health, Nantong University, Nantong, Jiangsu China

**Correction to: BMC Health Serv Res 21, 722 (2021).**

**https://doi.org/10.1186/s12913-021-06717-5**

Following publication of the original article [1], the order that the authors appeared in the author list was incorrect. The reason is that the authors mistakenly placed the corresponding author Qunhong Wu at first.

The author list has been updated above and the original article [1] has been corrected.
